# Blunt Head Trauma and Headache

**DOI:** 10.15844/pedneurbriefs-29-4-5

**Published:** 2015-04

**Authors:** Ana B. Chelse, Leon G. Epstein

**Affiliations:** 1 Division of Neurology, Ann & Robert H. Lurie Children's Hospital of Chicago, Chicago, IL; 2 Departments of Pediatrics and Neurology, Northwestern University Feinberg School of Medicine, Chicago, IL

**Keywords:** Blunt Head Trauma, Headache, Traumatic Brain Injury

## Abstract

Investigators from New York Presbyterian Morgan Stanley Children's Hospital examined whether having an isolated headache following minor blunt head trauma was suggestive of traumatic brain injury (TBI) among a large cohort of children 2-18 years of age.

Investigators from New York Presbyterian Morgan Stanley Children's Hospital examined whether having an isolated headache following minor blunt head trauma was suggestive of traumatic brain injury (TBI) among a large cohort of children 2-18 years of age [1]. This was a secondary analysis of data from a prospective observational multi-center cohort study from PECARN (Pediatric Emergency Care Applied Research Network). One group had an “extensive isolated” headache defined by GCS > 15 without loss of consciousness, altered mental status, signs of a basilar fracture, palpable skull fracture, vomiting, seizure, amnesia, scalp hematoma, or neurologic deficits. PECARN isolated headache was a second group [2]. Of 12, 567 children with headaches, 2,462 (19.6%) met the “extensive isolated” definition. CT scans were obtained on 52% of patients with headaches in general and in 18.5% of patients with isolated headache-extensive type. Clinically important TBI (ciTBI) occurred in none of the patients with isolated headaches and in 1.6% patients with non-isolated headaches at presentation. Three of 456 (0.7%) children with isolated headaches – extensive type had TBIs on head CT compared to 4.5% children with non-isolated headaches. After authors adjusted for mechanism of injury, children with isolated headaches–extensive type had significantly lower odds of TBI on CT compared to children with non-isolated headaches at presentation. One patient with PECARN isolated headache needed neurosurgery; however the child also had a temporal/parietal scalp hematoma on exam in addition to a severe headache. Severity, location, or timing of the headache was not found to correlate with ciTBI or TBI. Authors suggest that isolated headache following minor blunt head trauma is not suggestive of TBI in most children when no other symptoms are present. [1]

COMMENTARY. Head trauma in children results in 600,000 emergency room visits per year. The decision to obtain a CT must be balanced against its associated risks. Radiation from CT scans can cause malignancy in children and the risk has an inverse relationship with age. The PECARN prediction rules identified children at very low risk of ciTBI after blunt head trauma for whom CT scans might be avoided. Several studies report that less than 10% of CT scans show TBIs in children with minor head trauma [2, 3]. Additionally, in children with GCS scores >14, neurosurgical intervention is rarely needed [4]. Headache is a common complaint following head trauma [2–4]. Pandor et al. performed a meta-analysis on the presence of headache in children and adults. They found that the presence of a headache following minor head trauma, regardless of other symptoms was not associated with an increased overall risk of intracranial hemorrhage on CT; however, when headache was severe or persistent and accompanied other signs of TBI, it did modestly increase the risk of neurosurgery [5]. Headache alone in the PECARN study with no other symptoms had a 0.5% of TBI. The current study added to this knowledge by showing that no children had ciTBI following mild blunt head trauma. Further, TBIs on CT scans are very uncommon when isolated headache is the only symptom. The authors strongly suggest that CTs are not indicated in most children with isolated headache following minor blunt head trauma.
